# 6,6′-Dihydr­oxy-3,3′-dithio­dibenzoic acid

**DOI:** 10.1107/S1600536809023757

**Published:** 2009-06-27

**Authors:** Shu-Wen Sun, Fu-Lin Zhou, Gao-Feng Wang, Gui-Hua Cui

**Affiliations:** a College of Pharmacy, Jilin Medical College, Jilin 132013, People’s Republic of China; b Chemistry Department, Yuncheng University, Yuncheng, Shanxi 044000, People’s Republic of China

## Abstract

In the title compound, C_14_H_10_O_6_S_2_, the dihedral angle between the planes of the two phenyl­ene rings is 55.9 (1)°. Both hydr­oxy groups form intra­molecular hydrogen bonds; however, one of them also engages in inter­molecular hydrogen bonding. In the crystal, mol­ecules are connected into helical chains by O—H⋯O hydrogen bonds. The crystal studied was an inversion twin with a domain ratio of 0.51 (13):0.49 (13).

## Related literature

For hydrogen bonds and π–π stacking inter­actions in aromatic compounds, see: Janiak (2000 ▶); Hunter & Sanders (1990 ▶); Orr *et al.* (1999 ▶); Kaafarani *et al.* (2001 ▶). For a comparison of bond dimensions for disulfide compounds, see: Kaitner & Pavlovic (1997 ▶); Korp & Bernal (1984 ▶); Ni *et al.* (2004 ▶); Sacerdoti *et al.* (1975 ▶).
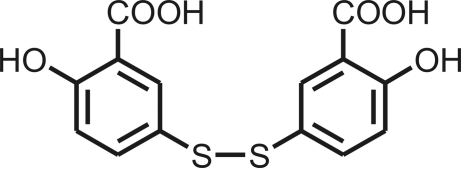


## Experimental

### 

#### Crystal data

C_14_H_10_O_6_S_2_
*M* *_r_* = 338.34
Orthorhombic, 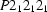

*a* = 5.3065 (6) Å
*b* = 11.1657 (13) Å
*c* = 23.906 (2) Å
*V* = 1416.5 (3) Å^3^
*Z* = 4
Mo *K*α radiation
μ = 0.40 mm^−1^
*T* = 298 K
0.24 × 0.15 × 0.14 mm

#### Data collection

Bruker SMART area-detector diffractometer
Absorption correction: multi-scan (*SADABS*; Sheldrick, 1996 ▶) *T*_min_ = 0.910, *T*_max_ = 0.946
6436 measured reflections
2502 independent reflections
2060 reflections with *I* > 2σ(*I*)
*R*_int_ = 0.043

#### Refinement

*R*[*F* ^2^ > 2σ(*F* ^2^)] = 0.045
*wR*(*F* ^2^) = 0.103
*S* = 1.09
2502 reflections
211 parameters
4 restraints
H atoms treated by a mixture of independent and constrained refinement
Δρ_max_ = 0.40 e Å^−3^
Δρ_min_ = −0.28 e Å^−3^
Absolute structure: Flack (1983 ▶), 1007 Friedel pairs
Flack parameter: 0.49 (13)

### 

Data collection: *SMART* (Bruker, 2004 ▶); cell refinement: *SAINT* (Bruker, 2004 ▶); data reduction: *SAINT*; program(s) used to solve structure: *SHELXS97* (Sheldrick, 2008 ▶); program(s) used to refine structure: *SHELXL97* (Sheldrick, 2008 ▶); molecular graphics: *SHELXTL* (Sheldrick, 2008 ▶); software used to prepare material for publication: *SHELXTL*.

## Supplementary Material

Crystal structure: contains datablocks I, global. DOI: 10.1107/S1600536809023757/ng2599sup1.cif

Structure factors: contains datablocks I. DOI: 10.1107/S1600536809023757/ng2599Isup2.hkl

Additional supplementary materials:  crystallographic information; 3D view; checkCIF report

## Figures and Tables

**Table 1:** Hydrogen-bond geometry (Å, °)

*D*—H⋯*A*	*D*—H	H⋯*A*	*D*⋯*A*	*D*—H⋯*A*
O2—H2⋯O5^i^	0.85 (3)	1.90 (3)	2.739 (4)	171 (4)
O3—H3⋯O1	0.84 (4)	1.91 (5)	2.616 (4)	142 (5)
O3—H3⋯O6^ii^	0.84 (4)	2.52 (4)	3.063 (5)	123 (4)
O4—H4⋯O1^iii^	0.85 (4)	1.79 (4)	2.636 (4)	175 (4)
O6—H6⋯O5	0.85 (4)	1.90 (4)	2.642 (4)	146 (5)

Symmetry codes: (i) 
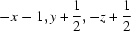
; (ii) 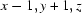; (iii) 
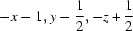
.

**Fig. 1.**
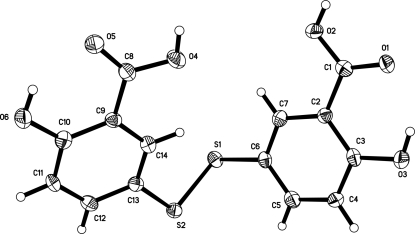
The molecular structure of (I), showing the atomic numbering. Displacement ellipsoids are drawn at the 30% probability level.

## supplementary crystallographic information

### Comment

Hydrogen bonds and π–π stacking interactions play an important role in the self-assembly and recognition of aromatic compounds((Janiak, 2000; Hunter & Sanders, 1990) as an auxiliary stabilizing short contact (Orr *et al.*, 1999; Kaafarani *et al.*, 2001).

The two phenyl rings of the title compound are bonded with two S atoms at a distance of 1.781 (4)Å (S2—C13) and 1.793 (4) Å (S1—C6), and the C—C distances of two phenyl groups range from 1.371 (5) to 1.407 (6) Å (Figure. 1 and Table 1). Interestingly, there are intermolecular S···S interactions in the crystal structures, which are not common in diphenyl disulfide derivaties (Korp & Bernal, 1984; Kaitner & Pavlovic 1997; Ni *et al.*, 2004; Sacerdoti *et al.*, 1975). The intermolecular S···S interactions distance is 3.414 (2) Å, whereas the shorter intramolecular S—S distance is 2.051 (2) Å. The dihedral angle of these two phenyl rings is 55.9 (1)°, which is different from other molecules such as 4, 4'-dithiodiphenol (48.1 (2)°) (Ni *et al.*, 2004) and diphenyl disulfide (76.7 (3)°) (Sacerdoti *et al.*, 1975).

The crystal structure of 5, 5'-dithiodisalicylic acid demonstrates the self-assembly of molecules into three dimensional networks *via* hydrogen bonds(Table 2) and intermolecular S···S interactions.

### Experimental

The title compound (I) was prepared as follows: To a solution of 5-(chlorosulfonyl)-2-hydroxybenzoic acid(19 mmol, 4.5 g) in conc. HCl (30 ml) cooled to 0 °C in an ice bath, Sn(118 mmol, 14.0 g) was added. The reaction mixture was stirred for 12 h and then refluxed for 6 h. The precipitate was separated and dissolved in Et_2_O. After filtration the organic layer was concentrated under reduced pressure to afford a solid that was subsequently purified by recrystallization using a mixture of EtOH and H_2_O. Yellow needle crystals of (I) were obtained by slow evaporation from EtOH/acetone/DMSO/DMF(5:1:3:3) after five months. Analysis calculated for C_14_H_10_O_6_S_2_: C 49.70, H 2.98%; found: C 49.18, H 2.75%.

### Refinement

All H atoms were placed in calculated positions and treated as riding, with C—H in the range 0.93–0.98 Å and with *U*_iso_(H)=1.2–1.5*U*_eq_(C).

### Crystal data

**Table:**

C_14_H_10_O_6_S_2_	*D*_x_ = 1.587 Mg m^−^^3^
*M**_r_* = 338.34	Melting point: 523 K
Orthorhombic, *P*2_1_2_1_2_1_	Mo *K*α radiation, λ = 0.71073 Å
Hall symbol: P 2ac 2ab	Cell parameters from 2489 reflections
*a* = 5.3065 (6) Å	θ = 2.5–25.5°
*b* = 11.1657 (13) Å	µ = 0.40 mm^−^^1^
*c* = 23.906 (2) Å	*T* = 298 K
*V* = 1416.5 (3) Å^3^	Needle, yellow
*Z* = 4	0.24 × 0.15 × 0.14 mm
*F*(000) = 696	

### Data collection

**Table:**

Bruker SMART area-detector diffractometer	2502 independent reflections
Radiation source: fine-focus sealed tube	2060 reflections with *I* > 2σ(*I*)
graphite	*R*_int_ = 0.043
φ and ω scans	θ_max_ = 25.0°, θ_min_ = 1.7°
Absorption correction: multi-scan (*SADABS*; Sheldrick, 1996)	*h* = −6→6
*T*_min_ = 0.910, *T*_max_ = 0.946	*k* = −13→13
6436 measured reflections	*l* = −28→12

### Refinement

**Table:**

Refinement on *F*^2^	Secondary atom site location: difference Fourier map
Least-squares matrix: full	Hydrogen site location: inferred from neighbouring sites
*R*[*F*^2^ > 2σ(*F*^2^)] = 0.045	H atoms treated by a mixture of independent and constrained refinement
*wR*(*F*^2^) = 0.103	*w* = 1/[σ^2^(*F*_o_^2^) + (0.0345*P*)^2^ + 0.9484*P*] where *P* = (*F*_o_^2^ + 2*F*_c_^2^)/3
*S* = 1.09	(Δ/σ)_max_ < 0.001
2502 reflections	Δρ_max_ = 0.40 e Å^−^^3^
211 parameters	Δρ_min_ = −0.28 e Å^−^^3^
4 restraints	Absolute structure: Flack (1983), 1007 Friedel pairs
Primary atom site location: structure-invariant direct methods	Flack parameter: 0.49 (13)

### Special details

**Table:**

Geometry. All e.s.d.'s (except the e.s.d. in the dihedral angle between two l.s. planes) are estimated using the full covariance matrix. The cell e.s.d.'s are taken into account individually in the estimation of e.s.d.'s in distances, angles and torsion angles; correlations between e.s.d.'s in cell parameters are only used when they are defined by crystal symmetry. An approximate (isotropic) treatment of cell e.s.d.'s is used for estimating e.s.d.'s involving l.s. planes.
Refinement. Refinement of *F*^2^ against ALL reflections. The weighted *R*-factor *wR* and goodness of fit *S* are based on *F*^2^, conventional *R*-factors *R* are based on *F*, with *F* set to zero for negative *F*^2^. The threshold expression of *F*^2^ > σ(*F*^2^) is used only for calculating *R*-factors(gt) *etc*. and is not relevant to the choice of reflections for refinement. *R*-factors based on *F*^2^ are statistically about twice as large as those based on *F*, and *R*- factors based on ALL data will be even larger.

### Fractional atomic coordinates and isotropic or equivalent isotropic displacement parameters (Å^2^)

**Table:**

	*x*	*y*	*z*	*U*_iso_*/*U*_eq_	
O1	−0.4934 (6)	0.8216 (2)	0.21892 (11)	0.0439 (7)	
O2	−0.6036 (6)	0.6399 (3)	0.18732 (11)	0.0439 (8)	
H2	−0.717 (6)	0.643 (4)	0.2123 (14)	0.066*	
O3	−0.1299 (6)	0.9391 (3)	0.16941 (13)	0.0519 (8)	
H3	−0.254 (6)	0.934 (5)	0.1911 (17)	0.078*	
O4	−0.1551 (7)	0.3528 (3)	0.20456 (12)	0.0505 (8)	
H4	−0.269 (7)	0.338 (4)	0.2286 (15)	0.076*	
O5	−0.0215 (6)	0.1743 (3)	0.23556 (11)	0.0438 (7)	
O6	0.3765 (6)	0.0759 (3)	0.18767 (12)	0.0503 (8)	
H6	0.252 (6)	0.080 (5)	0.2099 (17)	0.075*	
S1	−0.0244 (2)	0.52404 (9)	0.01641 (4)	0.0370 (3)	
S2	0.34345 (19)	0.46809 (9)	0.01989 (4)	0.0360 (2)	
C1	−0.4622 (8)	0.7376 (4)	0.18627 (14)	0.0343 (9)	
C2	−0.2645 (7)	0.7386 (3)	0.14288 (15)	0.0303 (9)	
C3	−0.1095 (8)	0.8396 (3)	0.13723 (15)	0.0360 (10)	
C4	0.0804 (8)	0.8402 (4)	0.09698 (16)	0.0391 (10)	
H4A	0.1880	0.9057	0.0942	0.047*	
C5	0.1095 (8)	0.7447 (3)	0.06154 (16)	0.0391 (10)	
H5	0.2337	0.7470	0.0341	0.047*	
C6	−0.0443 (8)	0.6441 (3)	0.06607 (15)	0.0324 (9)	
C7	−0.2280 (8)	0.6406 (3)	0.10693 (15)	0.0339 (9)	
H7	−0.3285	0.5729	0.1107	0.041*	
C8	−0.0058 (8)	0.2582 (4)	0.20199 (16)	0.0367 (9)	
C9	0.1795 (7)	0.2604 (3)	0.15645 (15)	0.0314 (9)	
C10	0.3593 (8)	0.1685 (3)	0.15118 (15)	0.0356 (9)	
C11	0.5317 (8)	0.1726 (3)	0.10729 (16)	0.0404 (10)	
H11	0.6534	0.1130	0.1041	0.048*	
C12	0.5236 (8)	0.2640 (3)	0.06854 (16)	0.0387 (9)	
H12	0.6396	0.2651	0.0394	0.046*	
C13	0.3440 (8)	0.3550 (3)	0.07246 (14)	0.0312 (9)	
C14	0.1790 (8)	0.3533 (3)	0.11681 (15)	0.0350 (9)	
H14	0.0637	0.4155	0.1207	0.042*	

### Atomic displacement parameters (Å^2^)

**Table:**

	*U*^11^	*U*^22^	*U*^33^	*U*^12^	*U*^13^	*U*^23^
O1	0.0515 (18)	0.0345 (16)	0.0456 (15)	0.0031 (16)	0.0185 (17)	−0.0067 (13)
O2	0.047 (2)	0.0394 (16)	0.0449 (16)	−0.0082 (15)	0.0179 (15)	−0.0035 (14)
O3	0.057 (2)	0.0356 (16)	0.063 (2)	−0.0068 (15)	0.0226 (17)	−0.0158 (15)
O4	0.0540 (19)	0.0474 (18)	0.0502 (18)	0.0124 (17)	0.0237 (16)	0.0074 (15)
O5	0.0474 (18)	0.0416 (17)	0.0423 (16)	0.0038 (16)	0.0113 (16)	0.0068 (13)
O6	0.050 (2)	0.0404 (17)	0.0605 (19)	0.0127 (16)	0.0138 (17)	0.0138 (16)
S1	0.0372 (6)	0.0428 (5)	0.0311 (5)	0.0055 (5)	−0.0017 (5)	−0.0050 (5)
S2	0.0371 (5)	0.0370 (5)	0.0340 (5)	0.0005 (5)	0.0064 (5)	0.0011 (5)
C1	0.034 (2)	0.040 (2)	0.0291 (19)	0.005 (2)	0.0003 (19)	0.0052 (18)
C2	0.031 (2)	0.0292 (19)	0.031 (2)	0.0008 (18)	−0.0014 (18)	0.0029 (17)
C3	0.040 (2)	0.032 (2)	0.036 (2)	0.0092 (19)	0.002 (2)	−0.0012 (18)
C4	0.036 (3)	0.033 (2)	0.049 (2)	−0.0035 (19)	0.006 (2)	0.004 (2)
C5	0.044 (3)	0.040 (2)	0.033 (2)	0.007 (2)	0.009 (2)	0.009 (2)
C6	0.036 (2)	0.031 (2)	0.0302 (19)	0.0064 (19)	−0.0022 (19)	0.0001 (16)
C7	0.039 (2)	0.031 (2)	0.032 (2)	0.0007 (19)	−0.0032 (19)	0.0034 (17)
C8	0.032 (2)	0.038 (2)	0.040 (2)	0.003 (2)	−0.001 (2)	−0.0059 (19)
C9	0.031 (2)	0.034 (2)	0.0297 (19)	−0.0025 (18)	0.0036 (18)	−0.0039 (17)
C10	0.041 (2)	0.034 (2)	0.032 (2)	0.000 (2)	−0.002 (2)	−0.0023 (17)
C11	0.034 (2)	0.034 (2)	0.052 (2)	0.011 (2)	0.009 (2)	−0.0032 (19)
C12	0.033 (2)	0.045 (2)	0.038 (2)	0.003 (2)	0.008 (2)	−0.0022 (19)
C13	0.031 (2)	0.033 (2)	0.0293 (19)	−0.0027 (19)	0.0005 (19)	−0.0065 (17)
C14	0.035 (2)	0.035 (2)	0.035 (2)	0.0044 (19)	−0.0009 (19)	−0.0021 (18)

### Geometric parameters (Å, °)

**Table:**

O1—C1	1.231 (4)	C4—C5	1.371 (5)
O2—C1	1.324 (5)	C4—H4A	0.9300
O2—H2	0.848 (10)	C5—C6	1.393 (5)
O3—C3	1.356 (5)	C5—H5	0.9300
O3—H3	0.842 (10)	C6—C7	1.380 (5)
O4—C8	1.322 (5)	C7—H7	0.9300
O4—H4	0.848 (10)	C8—C9	1.467 (5)
O5—C8	1.236 (5)	C9—C14	1.405 (5)
O6—C10	1.356 (4)	C9—C10	1.407 (6)
O6—H6	0.849 (10)	C10—C11	1.393 (5)
S1—C6	1.793 (4)	C11—C12	1.380 (5)
S1—S2	2.0511 (15)	C11—H11	0.9300
S2—C13	1.781 (4)	C12—C13	1.396 (5)
C1—C2	1.476 (5)	C12—H12	0.9300
C2—C3	1.402 (5)	C13—C14	1.375 (5)
C2—C7	1.404 (5)	C14—H14	0.9300
C3—C4	1.394 (5)		
			
C1—O2—H2	113 (3)	C6—C7—C2	120.6 (4)
C3—O3—H3	111 (4)	C6—C7—H7	119.7
C8—O4—H4	108 (4)	C2—C7—H7	119.7
C10—O6—H6	108 (4)	O5—C8—O4	122.4 (4)
C6—S1—S2	104.89 (14)	O5—C8—C9	122.7 (4)
C13—S2—S1	104.24 (14)	O4—C8—C9	115.0 (4)
O1—C1—O2	122.6 (4)	C14—C9—C10	118.6 (3)
O1—C1—C2	122.4 (4)	C14—C9—C8	120.8 (4)
O2—C1—C2	115.0 (3)	C10—C9—C8	120.6 (3)
C3—C2—C7	119.1 (3)	O6—C10—C11	117.7 (4)
C3—C2—C1	119.4 (3)	O6—C10—C9	123.0 (3)
C7—C2—C1	121.5 (3)	C11—C10—C9	119.3 (4)
O3—C3—C4	116.4 (4)	C12—C11—C10	120.6 (4)
O3—C3—C2	123.9 (4)	C12—C11—H11	119.7
C4—C3—C2	119.6 (4)	C10—C11—H11	119.7
C5—C4—C3	120.3 (4)	C11—C12—C13	121.0 (4)
C5—C4—H4A	119.9	C11—C12—H12	119.5
C3—C4—H4A	119.9	C13—C12—H12	119.5
C4—C5—C6	120.9 (4)	C14—C13—C12	118.4 (4)
C4—C5—H5	119.6	C14—C13—S2	123.6 (3)
C6—C5—H5	119.6	C12—C13—S2	118.0 (3)
C7—C6—C5	119.4 (3)	C13—C14—C9	121.9 (4)
C7—C6—S1	119.3 (3)	C13—C14—H14	119.0
C5—C6—S1	121.1 (3)	C9—C14—H14	119.0
			
C6—S1—S2—C13	89.96 (17)	O5—C8—C9—C14	175.4 (4)
O1—C1—C2—C3	−1.7 (5)	O4—C8—C9—C14	−4.7 (5)
O2—C1—C2—C3	178.1 (3)	O5—C8—C9—C10	−3.8 (6)
O1—C1—C2—C7	178.4 (4)	O4—C8—C9—C10	176.1 (4)
O2—C1—C2—C7	−1.7 (5)	C14—C9—C10—O6	179.2 (4)
C7—C2—C3—O3	179.5 (3)	C8—C9—C10—O6	−1.6 (6)
C1—C2—C3—O3	−0.3 (6)	C14—C9—C10—C11	0.4 (6)
C7—C2—C3—C4	−1.3 (6)	C8—C9—C10—C11	179.5 (4)
C1—C2—C3—C4	178.9 (3)	O6—C10—C11—C12	179.7 (4)
O3—C3—C4—C5	−178.1 (4)	C9—C10—C11—C12	−1.4 (6)
C2—C3—C4—C5	2.7 (6)	C10—C11—C12—C13	0.4 (6)
C3—C4—C5—C6	−1.9 (6)	C11—C12—C13—C14	1.8 (6)
C4—C5—C6—C7	−0.2 (6)	C11—C12—C13—S2	−178.8 (3)
C4—C5—C6—S1	174.9 (3)	S1—S2—C13—C14	−29.0 (3)
S2—S1—C6—C7	−127.2 (3)	S1—S2—C13—C12	151.6 (3)
S2—S1—C6—C5	57.7 (3)	C12—C13—C14—C9	−2.9 (6)
C5—C6—C7—C2	1.5 (6)	S2—C13—C14—C9	177.8 (3)
S1—C6—C7—C2	−173.7 (3)	C10—C9—C14—C13	1.8 (6)
C3—C2—C7—C6	−0.8 (6)	C8—C9—C14—C13	−177.4 (4)
C1—C2—C7—C6	179.0 (3)		

### Hydrogen-bond geometry (Å, °)

**Table:**

*D*—H···*A*	*D*—H	H···*A*	*D*···*A*	*D*—H···*A*
O2—H2···O5^i^	0.85 (3)	1.90 (3)	2.739 (4)	171 (4)
O3—H3···O1	0.84 (4)	1.91 (5)	2.616 (4)	142 (5)
O3—H3···O6^ii^	0.84 (4)	2.52 (4)	3.063 (5)	123 (4)
O4—H4···O1^iii^	0.85 (4)	1.79 (4)	2.636 (4)	175 (4)
O6—H6···O5	0.85 (4)	1.90 (4)	2.642 (4)	146 (5)

Symmetry codes: (i) −*x*−1, *y*+1/2, −*z*+1/2; (ii) *x*−1, *y*+1, *z*; (iii) −*x*−1, *y*−1/2, −*z*+1/2.
